# Original study on mathematical models for analysis of cellulose water content from absorbance/wavenumber shifts in ATR FT-IR spectrum

**DOI:** 10.1038/s41598-022-24097-6

**Published:** 2022-11-17

**Authors:** Stefan Cichosz, Anna Masek, Katarzyna Dems-Rudnicka

**Affiliations:** 1 Institute of Polymer and Dye Technology, Faculty of Chemistry, Lodz University of Technology, Stefanowskiego 16, 90-537 Lodz, Poland; 2 Centre of Mathematics and Physics, Lodz University of Technology, Politechniki 11, 90-924 Lodz, Poland

**Keywords:** Environmental sciences, Chemistry

## Abstract

The aim of this research was to evaluate the applicability of the attenuated total reflectance Fourier-transform infrared (ATR FT-IR) spectroscopy in the quantitative analysis of the moisture content in cellulose (from 0.5 to 11.0 wt.%). Innovatively, this work describes the variations in both absorbance and wavenumber of 16 absorption bands plotted as a function of cellulose water amount measured with Karl-Fischer titration. Different regression models were investigated (simple linear, semilogarithmic, power) and the adjusted coefficient of determination (R^2^) was given for each calculation. While model exhibited R^2^ > 90%, the standard error of calibration (SEC) was presented and an external validation has been performed. Regarding the absorbance-water content relationship, data recorded for sixteen peaks was successfully fitted with linear functions exhibiting R^2^ > 90%. The highest value of R^2^ = 98.7% and standard error of prediction SEP = 0.3wt.% have been assigned to the maximum from 3339 to 3327 cm^−1^ (–OH), proving ATR FT-IR usefulness in quantitative analysis.

## Introduction

Most commonly, time- and money-consuming techniques are employed for the determination of moisture content in natural fibres, e.g., thermogravimetric analysis (TGA)^1,2^ or Karl-Fischer titration^3,4^. Yet, in the recent years, attenuated total reflectance Fourier-transform infrared (ATR FT-IR) spectroscopy has been repeatedly proposed as a fast, cost-effective and non-invasive method in monitoring the moisture absorption processes in polymer-based systems^5–8^. Undoubtedly, this technique provides many attractive features, e.g., high sampling rate, sensitivity and the information at the molecular level contained in the vibrational spectra^9,10^. Therefore, FT-IR have been favourably used in quantitative description of cellulose chemical composition^11,12^ and investigation of hydrogen bonds^13,14^. Importantly, the development of attenuated total reflectance (ATR) FT-IR spectroscopy techniques enabled the use of this method directly onto solid materials^5,15^. Thus, making it easy to use in both industrial and scientific facilities. Below, some examples of the studies considering employment of ATR FT-IR spectroscopy technique in moisture content determination of cellulose-based materials have been discussed. Selected works favourably show different approaches in the analysis of water content in a biopolymer, both in terms of the applied statistical methods and the selection of IR spectrum regions for further investigation.

Firstly, Hou et al.^5^ have analysed the kinetics of water diffusion through the ethyl cellulose (EC) films. The spectral data has been collected as a function of time with acquisition interval of 40 s. The authors focused mainly on the region 3700–3100 cm^−1^ that is closely related to the O–H stretching vibration of water molecules^16^. It has been observed that the intensity of the O–H stretching band was gradually increasing in the first 2 h for all the investigated diffusion systems. Then, the researchers calculated the integrated areas of the O–H stretching region and plotted it as a function of diffusion time to obtain moisture diffusion curves. Moreover, to identify the functional groups participating in the water diffusion process, all the ATR FT-IR spectra at equal time intervals of 40 s were selected to carry out 2Dcos analysis. However, the 2D asynchronous maps were drawn only for the region 3700–3100 cm^−1^, thus revealing the spectral changes in a narrow sector of IR spectrum corresponding to the vibrations of hydroxyl moieties.

Similarly, Consumi et al.^6^ have come to the congruous conclusions. However, the authors used k-fold cross-validation technique based on the enlarged dataset of 80 samples divided into k = 5 folds. The researchers investigated carboxymethyl cellulose (CMC) of a varied moisture content and found that the intensities of the bands related to the stretching vibration of OH group (broad absorption band at 3392 cm^−1^ and a shoulder at 3255 cm^−1^) were significantly affected by the moisture presence. The authors determined the total area under the ATR FT-IR curve in the range 3675–2980 cm^−1^ and plotted it against the moisture content in cellulose sample. A simple linear regression including all the experimental data and IR band areas showed a good fit. Moreover, a satisfying correlation between the measured and predicted data was found for all the five training sets.

However, Célino et al.^7^ have concluded that not only a broad peak from 3700 to 3000 cm^−1^ might be useful in the quantitative analysis of water content in cellulose-based systems. The authors investigated the spectral signatures of various raw plant fibres, namely, hemp, flax and sisal at different moisture contents. The researchers described the molecular effect of water sorption mechanisms and employed a multivariate model linking different regions of FT-IR spectra. Firstly, the researchers performed Kruskal Wallis analysis on each individual wavenumber of the raw spectra. It highlighted several zones of the FT-IR spectrum that were strongly impacted by the increasing water uptake: (i) the broad band situated between 3600 and 3000 cm^−1^; (ii) the maximum at 1635 cm^−1^; (iii) the band placed in the wavenumber ranging from 1100 to 700 cm^−1^. Surprisingly, according to the Kruskal Wallis p-values assignment conducted by the researchers, the CH stretching band situated at 2935–2900 cm^−1^ was also found to be significantly impacted by the water uptake. Finally, the models were calculated promptly from the information contained in the spectral regions situated from 3600 to 3000 cm^−1^ and around 1650 cm^−1^.

Furthermore, Yuan et al.^8^ also tried to determine different IR regions useful in the quantitative analysis of the water content in cellulose-based systems. The researchers presented an interesting study on the moisture content determination in TEMPO oxidized cellulose nanocrystal film (TOCNF). The researchers concluded that primarily three spectrum ranges correlate with the moisture adsorption in cellulose-based materials: 3700–3000 cm^−1^, 1700–1580 cm^−1^ and 1180–1140 cm^−1^.

Contrary to the researches discussed above, this study relies on the data collected from 25-times-repeated moisture absorption/desorption experiments, hence, including the aspect of reproducibility, and employs a dedicated protocol of a mathematical analysis created for the purposes of the submitted research. Therefore, this study presents a statistic-based peak-by-peak investigation of both the absorbance and wavenumber shifts of 16 absorption bands visible in cellulose IR spectrum plotted as a function of moisture amount established with Karl-Fischer titration. Moreover, three regression models were investigated: simple linear, semilogarithmic, power. When the adjustment exhibited R^2^ > 90%, an external validation has been performed with ten additional IR-Fischer titration data packages. Consequently, the approach presented is a scientific novelty and the most cautious analysis done. Accordingly, this work undoubtedly provides the knowledge broadening on the IR spectrum employment possibilities as a non-destructive technique used in quantitative assessment of water content in cellulose.

## Materials and methods

### Materials

Cellulose fibres of a length between 6 and 12 µm, trade name Arbocel UFC100 Ultrafine Cellulose for Paper and Board Coating, with a density referred as 1.3 g/cm^3^ was delivered by J. Rettenmaier & Söhne (Rosenberg, Germany). Its specific surface area has been established at 3.9 m^2^/g and the total pore volume—0.02 cm^2^/g. Crystallinity index of cellulose measured with wide angle X-ray scattering (WAXS) is approximately 60%. Phosphorus oxide (V) and potassium nitrate were purchased from Chempur (Poland, Piekary Slaskie) and employed as the desiccator cartridges for, respectively, cellulose conditioning and moisture absorption experiment. Phosphorous oxide (V) is a solid that exhibits pH of approximately 1.5 and density of about 2.3 g/cm^3^. It is referred to provide an atmosphere of the relative humidity (RH) of approximately 0%^17^. In turn, the solubility of potassium nitrate in water is on the level of 316 g/L (20 °C). According to literature, its saturated aqueous solution is able to provide the atmosphere of a specific relative humidity which is approximately 96%^17^. Additionally, Hydranal Solvent E and Hydranal Titrant 5E, used during Karl-Fischer titration experiment, were supplied by Honeywell Fluka (Loughborough, UK).

## Methods

### Preparation of cellulose specimens of a varied moisture content

Cellulose studied in this research has been put into separate vessels: 0.1 g (Fourier-transform infrared spectroscopy) and 1.5 g (Karl Fischer titration) of cellulose in each vessel. Next, cellulose conditioning, moisture absorption and desorption experiments were caried out: (i) cellulose conditioning in the desiccator filled with phosphorus oxide (RH = 0%) for 7 days (all prepared weighing bottles with cellulose); (ii) moisture absorption experiment: desiccator filled with saturated solution of potassium nitrate; experiment lasted 24 h, measurements after 0, 1, 3, 5, 8, 24 h (the last measurement after 24 h is the beginning of the moisture desorption stage at 0 h); (iii) moisture desorption experiment: laboratory dryer (Binder, Tuttlingen, Germany) at 100 °C for 8 h, measurements after 0, 1, 3, 5, 8 h. To obtain reliable results, the described above process of moisture absorption/desorption by cellulose was repeated 25 times. Then, the amount of n = 250 spectra were recorded.

### Determination of cellulose properties

#### Fourier-transform infrared spectroscopy

Fourier transform infrared spectroscopy (FT-IR) absorbance spectra were recorded within the 4000–400 cm^−1^ range. To ensure an acceptable signal-to-noise ratio, 64 scans at resolution of 4 cm^−1^ were accumulated (absorption mode). The experiment has been performed with the use of Thermo Scientific Nicolet 6700 FT-IR spectrometer equipped with diamond Smart Orbit iTX attenuated total reflection (ATR) sampling accessory (Waltham, MA, USA). All the spectra were recorded to the background spectra. The measurements were carried out immediately after taking certain cellulose sample out of a desiccator/dryer (depending on the experiment stage). Firstly, the background spectrum was recorded. Then, the specimen has been put directly onto the crystal in ATR accessory and the recorded spectra were baseline corrected with OMNIC 9.2.86 software by selecting the lowest point on each spectrum and drawing a tangent to that point. The minimal resolution of the spectrometer is 0.5 cm^−1^. Moreover, the behaviour of the peaks recorded with ATR FT-IR technique might differ regarding the penetration depth of the radiation. According to Eq. (1)^18^, the penetration depth (${d}_{p}$) raises from 0.49 µm at 4000 cm^−1^ to 4.91 µm at 400 cm^−1^, while assuming: $\lambda$—the infrared wavelength (${\lambda }_{4000 {\mathrm{cm}}^{-1}}$ = 2.5 µm and ${\lambda }_{400 {\mathrm{cm}}^{-1}}$  = 25 µm), ${n}_{1}$ is the refractive index of the internal reflection element (${n}_{1}$= 2.41)^18^, ${n}_{2}$ is the refractive index of the substance investigated (${n}_{2}$ = 1.5)^19^, $\Theta$ is the incidence angle of IR beam ($\Theta$ = 45°).

1 $${d}_{p}=\frac{\lambda }{2\pi {n}_{1}\sqrt{{\mathrm{sin}}^{2}\Theta -{\left(\frac{{n}_{2}}{{n}_{1}}\right)}^{2}}}$$

Depending on the penetration depth of radiation at different wavelengths, the chemical and physical interactions at distinct levels could be observed. Therefore, the signals interpretation may vary depending on the wavenumber assigned to a certain maximum, e.g., chemical groups visible in IR spectrum from 4000 to 3000 cm^−1^ originate from the surface layer of the fibre, while moieties evidenced in the region from 1000 to 400 cm^−1^ might be placed deeper under the cellulose fibre surface. It is likely that the chemical moieties inside the cellulose fibres would interact differently with the solvent compared to the groups situated on the surface.

#### Karl-Fischer titration

At the same time as FT-IR experiment, Karl-Fischer titration was carried out with the use of TitroLine Alpha (Schott, Mainz, Germany) device. For each experiment approximately 1.3 g of cellulose sample and 30 ml of Hydranal Solvent E were taken. Hydrantal Titrant 5E was used as the titrant, while Hydranal Solvent E was used in the form of the solution into which the analyte was introduced. First, the amount of water in Hydranal Solvent E was determined. This amount was automatically subtracted by the operating system of the apparatus. Then, the cellulose sample was taken out of the desiccator/dryer and the exact sample was weighed. Later, a cellulose specimen was introduced into the vessel of the apparatus and the measurement was performed. End-point detection has been performed while the potential recorded was unchanged for more than 10 s. The titrant concentration was determined every five measurements.

#### Data analysis

**Figure 1:**
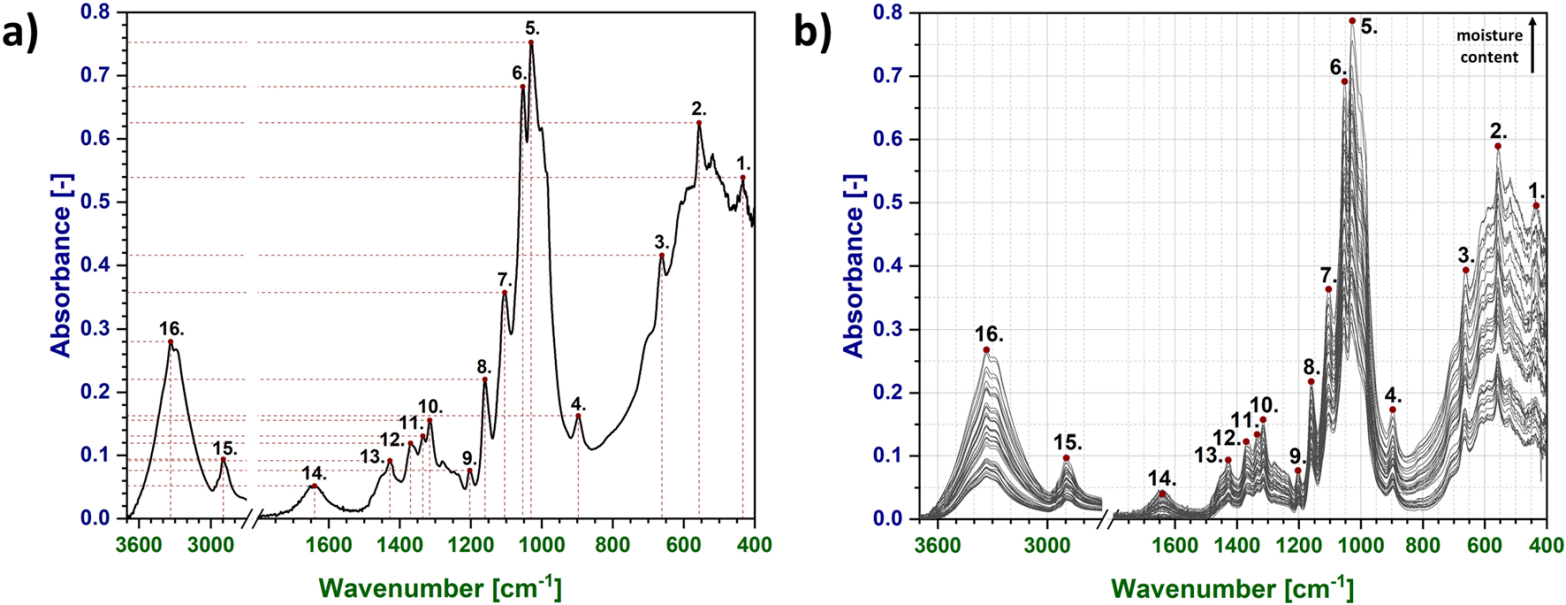
Exemplary ATR FT-IR spectrum of cellulose with the assignment of the peaks analysed (**a**) and visualization of the changes witnessed in the IR spectra with the elevating moisture content (**b**). Throughout the whole manuscript absorbance values are marked with dark blue and wavenumber values with dark green.

Coordinates (absorbance, wavenumber) of the selected peaks from IR spectra were read from .csv files (with the function of MS Excel searching for maxima in selected spectral regions; available in Supplementary file) and plotted as a function of moisture content in cellulose fibres determined with Karl-Fischer titration. Absorption bands taken into consideration in this study are presented in Fig. 1a. Sixteen selected maxima have been marked with red dots. Each red dot represents a point regarded for further mathematical analysis and the dashed lines reveal how the coordinates (X—wavenumber, Y—absorbance) have been read. The total amount of investigated cellulose samples is n = 250 (entire numerical data gathered in this study has been presented in Supplementary file).

Additionally, Table 1 gathers information about a chemical meaning behind the selected absorption bands and reveals the assignment of sixteen maxima to the chemical moieties present in the structure of investigated biopolymer. Further information on the interpretation of cellulose IR spectrum is available elsewhere^16,20^.

**Table 1:** Assignment of the analysed absorption bands to chemical moieties embodied in cellulose structure.

Peak no	Wavenumber range (cm^−1^)	Chemical group	References
1	444–432	Vibration of C–O bonds	^22^
2	560–556	γCH, characteristic of cellulose I	^23^
3	668–660	δCOH out of plane	^13^
4	900–895	Asymmetric γCOC at β-glycosidic bond; amorphous region	^24^
5	1032–1027	γCO assymetric deformation at C6	^13^
6	1056–1051	γCO stretching at C3	^13^
7	1107–1103	γ ring in plane	^25^
8	1162–1159	γCOC at β-glycosidic linkage, symmetric	^24^
9	1203–1199	δCOH in plane, symmetric	^24^
10	1316–1314	δCH_2_ (wagging), symmetric	^26^
11	1335–1332	δCOH in plane, asymmetric	^27^
12	1370–1360	Tertiary δCH	^28^
13	1429–1425	δCH_2_ (symmetric); crystalline region	^24^
14	1646–1636	Absorbed water; –OH bending vibration	^24^
15	2898–2888	Asymmetric γCH (*sp*^3^)	^29^
16	3339–3327	Associative γOH bond, hydrogen bonding	^16^

Then, RStudio (version 1.4.1106) software was employed in data analysis^21^. The data was imported into the program, and then the analysis of individual linear models was performed along with the assessment of their adequacy using the program's built-in functions. Tree models, namely, simple linear (Eq. 2), semilogarithmic (Eq. 3), power (Eq. 4), were investigated to find the relationship between the variables:

2 $$Y=a\cdot X+b$$

3 $$Y=a\cdot \mathrm{ln}\left(X\right)+b$$

4 $$Y={a}_{1}{X}^{{b}_{1}}\Leftrightarrow \mathrm{ln}\left(Y\right)=a\cdot \mathrm{ln}\left(X\right)+b$$

$Y$ refers to a measured parameter (absorbance or wavenumber) read from IR spectrum and $X$ is a moisture content. First, the simple linear model was assessed. If the analysed data did not show a linear relationship, two successive models (semilogarithmic, power) were examined. The quality of the models was estimated using the adjusted coefficient of determination (R^2^) and standard error value for calibration (SEC). Additionally, an independent data set used for the model external validation (10 samples of recorded IR spectrum and determined moisture content that were not taken into consideration during the calibration of the models) has been employed to assess prediction adequacy with adjusted coefficient of determination and standard error for prediction (SEP). For a reliable model, the R^2^ values should be close to 100% (or 1), while SEC and SEP values should be as low as possible.

Moreover, according to the model described by Eq. (5), a multivariate multiple regression has been applied in the analysis of cellulose moisture content-absorbance relationship:

5 $$Y={\beta }_{0}+{\beta }_{1}{X}_{1}+{\beta }_{2}{X}_{2}+\dots +{\beta }_{k}{X}_{k}$$

where ${X}_{1}$, ${X}_{2}$, …, ${X}_{k}$ reflect the absorbance values recorded for $k$ = 16 peaks, $Y$ refers to the predicted value of a moisture content and ${\beta }_{1}$, ${\beta }_{2}$, …, ${\beta }_{k}$ are model parameters (regression coefficients). The results obtained with the multivariate model were subsequently compared with the values recorded from Karl-Fischer titration experiment in the way described for previous mathematical models. Graphs presented in this research were prepared using *ggplot2* package from the RStudio (version 1.4.1106) software. Moreover, linear models have been established with the following commands: simple linear—model <- lm($Y$ ~ $X$, data = data_file), semilogarithmic—model <- lm($Y$ ~ log($X$), data = data_file), power—model <- lm(log($Y$) ~ log($X$), data = data_file).

### Ethics approval and consent to participate

All authors approve the submitted version of the manuscript and will to participate.

## Results and discussion

### Linear models assessed

In this study, the variations of X- and Y-axis coordinates of sixteen selected peaks were investigated and plotted as a function of moisture content. This means, not the changes in the spectrum heigh at the specified and fixed positions were measured (Fig. 1b), but the cautious evaluation on the real maxima coordinates of sixteen selected peaks has been conducted. Therefore, the approach presented relevantly differs from previously performed studies^5–8^. It accounts the movements of the absorption bands’ maxima along both the axes of frequency and absorbance, as well as considers sixteen different peaks attributed to chemical groups embodied in cellulose structure.

Furtherly, as every cellulose IR analysis performed was accompanied by a short and precise Karl-Fischer titration experiment, the moisture content of each biopolymer sample has been successfully established. Then, the coordinates of absorption bands’ maxima were plotted as a function of water content in cellulose specimens. Consequently, to reduce the studied dependencies to a linear form, the regression adjustment has been performed separately for each peak. The results of the carried out investigation for three different models (simple linear, semilogarithmic, power) have been gathered in Table 2. The adjusted coefficient of determination (R^2^) was given for each model analysed. Furthermore, in case of absorbance-based models that exhibited R^2^ > 90% the standard error of calibration (SEC) was presented as well. Importantly, an external validation has been performed with ten additional IR-Fischer titration data packages.

**Table 2:** Quality parameters for models’ calibration and external set validation for the models selected (p-value for each regression on the level < 0.5).

Peak no	Absorbance-based simple linear model				Wavenumber-based calibration		
	Calibration		External validation				
	SEC (–)	R^2^ (%)	SEP (wt.%)	R^2^ (%)	Simple linear model R^2^ (%)	Semilogarithmic model R^2^ (%)	Power model R^2^ (%)
1	0.02	96.9	0.5	99.2	10.6	10.2	10.2
2	0.02	96.3	0.5	99.1	70.0	75.6	75.6
3	0.02	96.8	0.5	99.1	65.7	64.9	64.9
4	0.008	95.9	0.4	99.5	13.4	9.8	9.8
5	0.04	92.0	0.6	98.8	41.4	46.4	46.4
6	0.04	92.8	0.6	98.9	64.0	69.0	69.0
7	0.02	94.0	0.5	99.1	9.6	16.4	16.4
8	0.009	95.6	0.5	99.3	30.1	50.3	50.3
9	0.004	93.1	0.5	99.3	66.4	66.7	66.7
10	0.006	97.0	0.4	99.5	4.8	3.7	3.7
11	0.005	96.5	0.4	99.5	45.4	51.2	51.2
12	0.005	96.4	0.4	99.5	41.8	48.6	48.5
13	0.004	96.3	0.4	99.5	46.4	50.5	50.5
14	0.005	92.6	0.7	98.3	2.3	1.8	1.8
15	0.005	94.9	0.5	99.2	58.3	49.0	49.0
16	0.008	98.7	0.3	99.8	6.1	8.0	8.0

*SEC* standard error of calibration, *SEP* standard error of prediction, *R*^*2*^ adjusted coefficient of determination.

From the information presented in Table 2, it is clearly visible that ATR FT-IR technique has a great potential as a tool in quantitative determination of water content in cellulose. It might be concluded that the absorbance-based simple linear model favourably described the gathered data, because R^2^ assigned to the simple linear fits for all peaks investigated exhibited a value higher than 90%. Therefore, the remaining semilogarithmic and power models have not been taken into consideration. Yet, regarding the wavenumber-based models, all three fits were investigated and none of them exhibited a value of R^2^ higher than 90%, hence, they did not describe the data with a sufficient reliability. Further description of the models analysed in this study, as well as the observed changes of the peaks’ coordinates with raising moisture content of cellulose have been presented in the subsequent sections of the article.

### Absorbance-moisture content dependency

**Figure 2:**
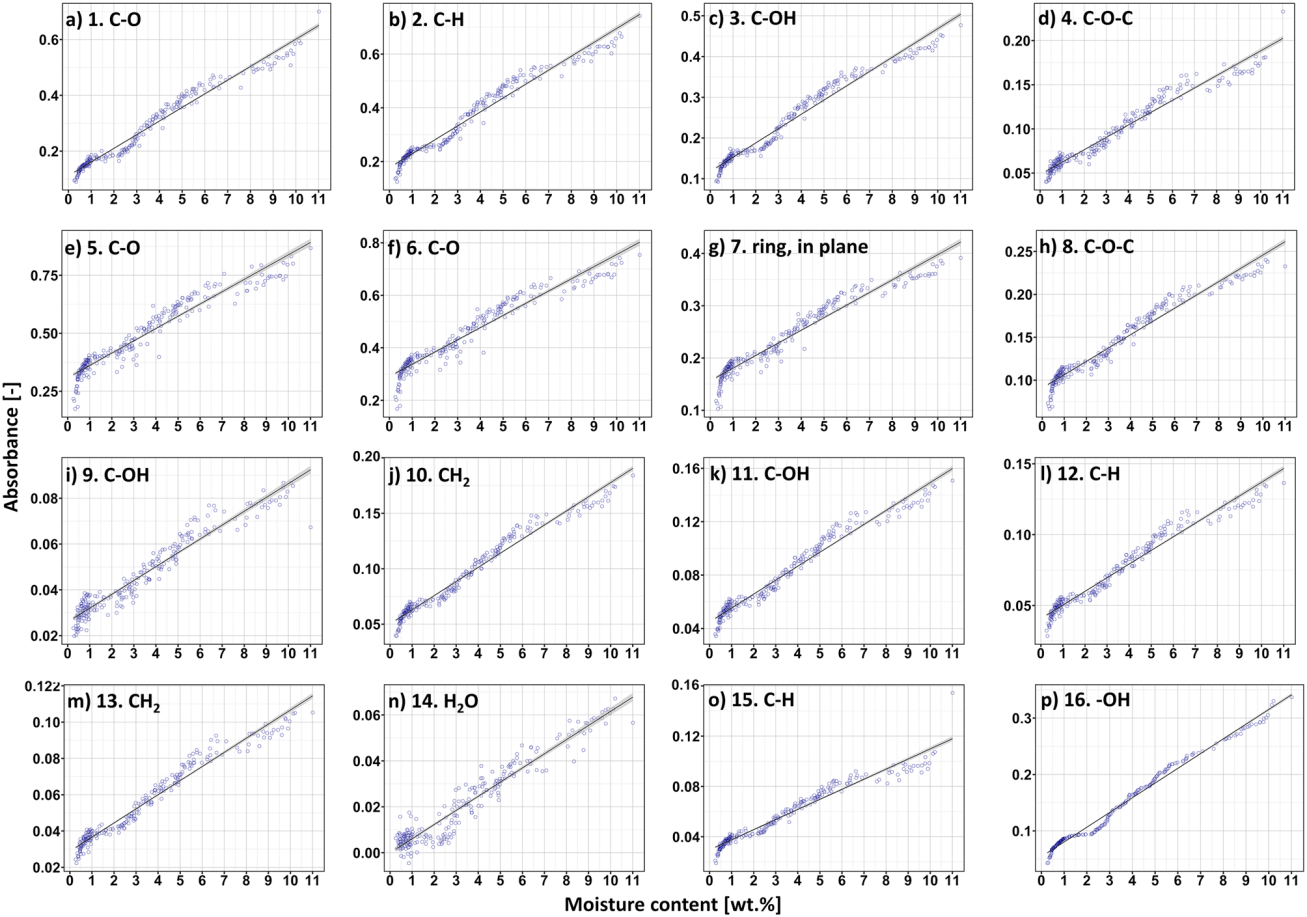
Height of the selected peaks (**a–p**) plotted as a function of moisture content in cellulose fibres determined through Karl-Fischer titration. Experimentally measured band absorbance (dark blue) and mathematically adjusted regression (black) with marked confidence area (grey).

Figure 2 reveals the experimental data regarding the changes in the absorbance values (height of the peak) assigned to sixteen maxima visible in IR spectrum of cellulose fibres. Additionally, the black lines represent the regression functions that coefficients of determination (R^2^) and standard errors of calibration (SEC) have been shown in Table 2.

Firstly, giving a closer look at Fig. 2, in all 16 cases, the absorbance is lower around moisture content of approx. 2wt.%, then higher from 3 to 8wt.%, and again lower than predicted by the linear model. This might be caused by an uncontrolled re-equilibration process before carrying out new measurement. For a short time, the specimens are exposed to ambient conditions before each recording, and that this allows a re-equilibration (and thus deviation) with the ambient humidity. The other possibility could be the sensitiveness of the selected analytical techniques for the slight changes in cellulose moisture content.

However, from Fig. 2 it might be also easily perceived that absorbance of all 16 peaks analysed is changing significantly while the moisture content in cellulose specimens elevates. The similar phenomenon has been observed by some other scientists^5–8^. However, in this study the raise in the height of each peak recorded have been successfully described with linear functions exhibiting R^2^ > 90%. High value of adjusted determination coefficient means the models applied reliably reflected the experimental data.

Interestingly, signals originated from both oxygen-embodying (peaks no.: 1, 3, 4, 5, 6, 8, 9, 11, 16) and oxygen-lacking (peaks no.: 2, 7, 10, 12, 13, 15) chemical groups responded to the variations in moisture content in cellulose fibres. It is not surprising that changes in the maxima assigned to oxygen-rich chemical moieties, being able to form hydrogen bonds with water molecules, altered during the water absorption process^30^. Because of the changes in cellulose-water interactions, the investigated chemical groups vibrate differently and differently react to the infrared radiation. Hence, absorbance/wavenumber shifts of the peaks could have become visible in IR spectrum (peaks no.: 1, 3, 4, 5, 6, 8, 9, 11, 16).

However, it is not certain what kind of mechanism stands behind the recorded variations in absorbance/wavenumber of the peaks assigned to non-polar CH_2_ and C-H chemical groups that are not able to directly interact with water molecules (peaks no.: 2, 7, 10, 12, 13, 15). Nevertheless, Celino et al.^7^ proposed a theory that could bring some elements of understanding. The changes observed might be favourably described with the surrounding signals originated from oxygen-embodying chemical moieties. The shoulders of the maxima assigned to carbon- and oxygen-rich chemical groups overlap, thus, affecting the shape, as well as the height of the peaks visible in IR spectrum.

Another explanation has been proposed by Yuan et al.^8^ who concluded that IR spectrum might also react to the cellulose chain stiffening caused by moisture absorption. The scientists referred that the changes in the macromolecule’s stiffness might contribute to the shifts along both absorbance and frequency axes (due to the differences in the strength of a certain bonding).

Furtherly, the attention should be drawn to some interesting results that have been shown in Fig. 2n. Peak no. 14 is generally assigned to the moisture absorbed in cellulose (water content)^24^. Importantly, this maximum is not present in IR spectrum of the biopolymer in a dried state. Therefore, its appearance could be only related to the water absorbed by cellulose fibres. Consequently, it should precisely reflect the amount of moisture bonded to cellulose. However, an opposite effect could have been observed. Peak no. 14 exhibits one of the lowest values of adjusted determination coefficient R^2^ = 92.6% and the highest standard error of calibration SEC = 0.05 (Table 2). This observation could be explained with the nature of the peak. This spectral range is fragile to the atmospheric moisture^31^. Hence, it is difficult to describe changes in the absorbance of peak no. 14 with a mathematic model because of the moisture present in the air. However, this test could be repeated in a moisture-free atmosphere. Probably, the obtained results would be more reliable. Nevertheless, such a complex measuring system would not find much use in practice.

Furtherly, maxima visible in IR spectrum are the common effect of many smaller signals that overlap. Therefore, their position and height are determined by many different interactions^11,32,33^, e.g. hydrogen bonds^34^, van der Waals forces^35^. Theoretically, these interactions may stabilize the peak’s position. If assume that peaks consisting of multiple signals and separated from the remaining absorption bands (restricted overlapping with neighbouring maxima) describe water uptake more reliably, a mathematical model assigned to an alone absorption band that might be deconvolved into different signals (especially corresponding to water-cellulose hydrogen bonds) should more accurately describe the changes in cellulose moisture content. Interestingly, this theory has found a confirmation in practice. The highest value of the adjusted coefficient of determination (R^2^ = 98.7%) has been recorded for partially separated peak no. 16 that could be successfully deconvoluted into three types of hydrogen bonding: intramolecular, intermolecular^16,33^. Most likely, the position of this peak is stabilized with three species of interactions that might be affected by the presence of water molecules. Then, the outcome is a describable and observable peak’s shift along the Y axis.

**Figure 3:**
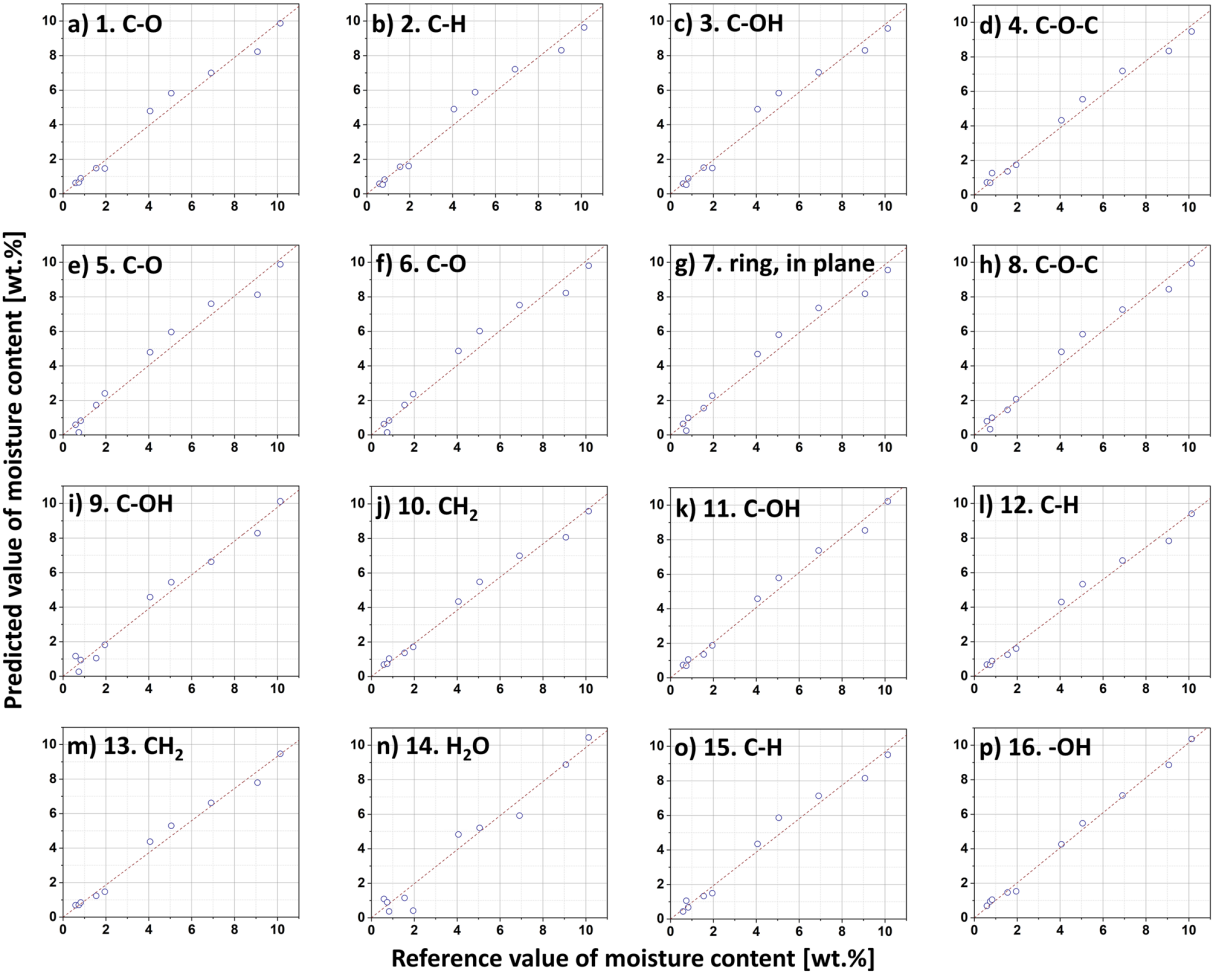
External validation results of water content prediction for cellulose fibres. The predictions of the moisture content obtained with the ATR FT-IR approach were compared to the Karl-Fischer titration measurements.

Next, all investigated models have been validated with external experimental data set. The results of the validation performed are presented in Table 2 and Fig. 3. The mathematical functions developed in this study revealed the satisfactory similarity between the newly recorded data and the values predicted with the linear functions. The coefficients of determination for validated models exhibited values from 98.3 to 99.8% with the best fit for peak no. 16.

However, it would be also worth considering whether it is reasonable to use only one absorption band to determine the water content of natural cellulose fibres, or whether the results calculated from the different absorption bands should be averaged. Therefore, additional calculations were made. The determined moisture contents obtained from the 16 absorption bands were averaged for each experimental point. Then, external validation was performed. The adjusted coefficient of determination was at the level of R^2^ = 99.5%, and the standard SEP prediction error—SEP = 0.4wt.%. These values indicate a slightly lower accuracy of such a method along with the determination of the moisture content in cellulose for, e.g., peak no. 16. Thus, proving once again the accuracy of the determinations made using this absorption band.

Additionally, a multivariate multiple regression analysis has been performed. This approach allowed to determine the relationship between the moisture content of cellulose fibres and the absorbance of multiple peaks recorded in IR spectrum. The results of the performed analysis have been presented in Table 3. It is visible that the mathematical model proposed has been characterized by the good correlation between the variables (coefficient of determination for calibration R^2^ of approx. 99%) and the lowest recorded SEP = 0.2wt.%, indicating the sufficient and reliable description of the data analysed. Moreover, peak no. 16, with the p-value < 0.001, showed the highest significance in the investigation performed. This means, it might be favourably used alone in cellulose moisture content analysis.

**Table 3:** Quality parameters for multivariate multiple regression.

Peak no	Coefficients ${\upbeta }_{\mathrm{k}}$ (−)	Significance (p-value)	Multivariate model statistics			
			Calibration		External validation	
			SEC (wt.%)	R^2^ (%)	SEP (wt.%)	R^2^ (%)
-	${\beta }_{0}$= −1.2	***				
1	${\beta }_{1}$= 3.5	–	0.3	98.9	0.2	99.5
2	${\beta }_{2}$= −4.4	–				
3	${\beta }_{3}$= 5.0	–				
4	${\beta }_{4}$= −4.8	–				
5	${\beta }_{5}$= 8.7	–				
6	${\beta }_{6}$= −25.5	–				
7	${\beta }_{7}$= 77.4	**				
8	${\beta }_{8}$= −84.9	*				
9	${\beta }_{9}$= −6.5	–				
10	${\beta }_{10}$= −171.6	*				
11	${\beta }_{11}$= 209.6	–				
12	${\beta }_{12}$= −37.9	–				
13	${\beta }_{13}$= −2.5	–				
14	${\beta }_{14}$= 32.1	*				
15	${\beta }_{15}$= −9.5	–				
16	${\beta }_{16}$= 54.0	***				

*SEC* standard error of calibration, *SEP* standard error of prediction, *R*^*2*^ adjusted coefficient of determination.

p-value: > 0.1, *< 0.05, **< 0.01, ***< 0.001.

The data collected above undoubtedly indicates the information collected with ATR FT-IR technique could be successfully correlated with volumetric measurements of moisture content in cellulose (e.g., Karl-Fischer titration). Consequently, ATR FT-IR method might be favourably regarded as a quantitative technique for the determination of moisture content in cellulose fibres.

### Wavenumber-moisture content relationship

**Figure 4:**
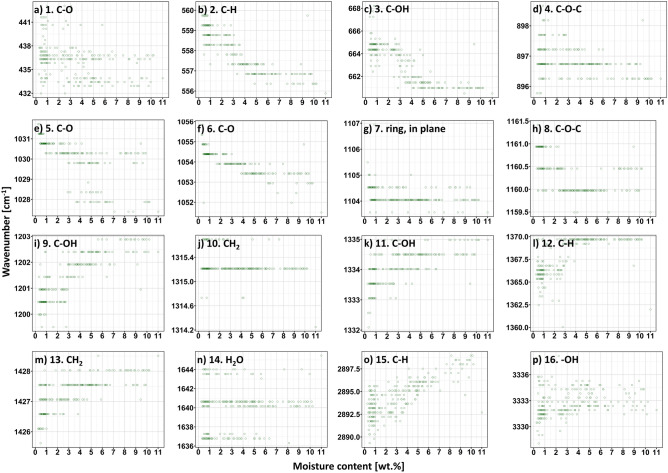
Wavenumber of the selected peaks (**a–p**) plotted as a function of moisture content in cellulose fibres determined by the means of Karl-Fischer titration. No linear regressions were found.

Additionally, this is the first study that have deeply investigated the wavenumber-moisture content relationship and the possibility of the mathematical models adjustment^5–8^. The gathered experimental points plotted as a function of moisture content in cellulose have been shown in Fig. 4. Similarly, as in the previous subsection, the dots reflect the recorded data plotted as a function of moisture content. However, this time, adjusting of any regression was not possible. According to the data presented in Table 2, none of the investigated models (linear, semilogarithmic, power) did exhibit a sufficient value of adjusted determination coefficient (R^2^ < 90%), hence, indicating inaccurate description of experimental data.

Although the linear regression models were unable to sufficiently reflect the collected experimental points, the data presented in Fig. 4 contains some important scientific information and deserves a brief discussion. It might be easily noticed that the behaviour of the maxima differs. Some peaks shift along X axis (peaks no.: 2, 3, 4, 6, 9, 11, 12, 13, 15), while the others remain relatively unchanged (peaks no.: 7, 10, 16) or exhibit specified positions irrespective of the moisture content (peaks no.: 1, 8, 14).

Most likely, the shifts described above might be related to the water-cellulose intermolecular interactions and different responses of the specified chemical groups to these attraction forces^36,37^. To better describe these relationships, it is worth to analyse the following equation (Eq. 6) that determines the frequency of vibrations of chemical groups $\nu$ in IR spectrum (based on the spring action)^38^:

6 $$\nu =\frac{1}{2\pi \cdot c}\sqrt{\frac{k}{\mu }}$$

where: $c$ is the speed of light ($c=2.998\cdot {10}^{10}\frac{cm}{s}$), $k$ is the force constant characteristic of the bonding [kg/s^2^], $\mu$ is the reduced mass ($\mu =\frac{{m}_{1}{m}_{2}}{{m}_{1}+{m}_{2}}$; ${m}_{i}$ is the molar mass of the atom $i$) [−]. Since the shifts of absorption bands assigned to the same chemical groups are investigated, $\mu$ remains constant (two atoms taking part in the bond formation are the same), c and $2\pi$ are also constant, hence, for any higher $k$ the $\nu$ is increased. Therefore, a bond characterised by a higher constant $k$ exhibits an elevated IR frequency when comparing the same type of vibrational motion. In other words, the higher force constant $k$ means a stiffer "spring", namely, stronger bond.

Consequently, the absorption bands that change their position with increasing moisture content are the most interesting and informative considering the determination of active sites capable of water bonding. The peaks’ shift to the lower wavenumbers (peaks no.: 2, 3, 4, 6), purely theoretically, indicate a weakening of the bond strength^38^ in these chemical moieties during the moisture absorption process, while moving of the peaks no.: 9, 11, 12, 13, 15 toward higher values of frequency indicates possible bond stiffening/strengthening.

Obviously, the signals mentioned above are mostly assigned to the polar moieties (able to form hydrogen bonds with water molecules^39^), e.g., peaks no. 3, 4, 6, 9, 11. However, neighbouring to them maxima attributed to non-polar groups (peaks no. 2, 12, 13, 15) also shift significantly. This might be caused by the overlapping with the signals originated from polar moieties^40^ or chain stiffening (hence, affecting the force constant $k$) during the water absorption process.

## Conclusions

The variations of both coordinates, absorbance and wavenumber, of sixteen peaks visible in cellulose ATR FT-IR spectrum were successfully investigated and plotted as a function of the moisture content (0.5-11wt.%) established with Karl-Fischer titration. Regarding the absorbance-water content relationship, data recorded for all considered maxima was fitted with mathematical models exhibiting adjustment of R^2^ > 90% and standard error of prediction approx. 0.4wt.%. Furthermore, absorption bands that wavenumber shifts could provide significant information on the behaviour of the active centres in cellulose have been favourably defined. Hence, proving ATR FT-IR applicability in both quantitative and qualitative analysis of cellulose water content.

## Future perspectives

Regarding absorbance-moisture content models, it should be determined in the future how the changes in crystallinity, content of hemicelluloses/lignin might affect the models established. Only then, the utility of the presented mathematical functions in determination of cellulose moisture content could be fully assessed. In turn, considering wavenumber-moisture content relationships, band displacements are being often studied under idealized conditions in a gaseous or liquid non-polar solvents^41,42^ yielding the shifts at the higher levels. Therefore, further investigation with a changed methodology is required.

## Supplementary Information

Supplementary Tables.

## Data Availability

Data gathered during this research has been presented in Supplementary file.
